# miR-497-5p/SALL4 axis promotes stemness phenotype of choriocarcinoma and forms a feedback loop with DNMT-mediated epigenetic regulation

**DOI:** 10.1038/s41419-021-04315-1

**Published:** 2021-11-03

**Authors:** Zheng Peng, Yi Zhang, Dazun Shi, Yanyan Jia, Huirong Shi, Huining Liu

**Affiliations:** 1 Department of Gynecology and Obstetrics, The First Affiliated Hospital of Zhengzhou University, Zhengzhou, 450052 China; 2 Department of Gynecology and Obstetrics, Xiangya Hospital of Central South University, Changsha, 410008 China

**Keywords:** Oncogenes, miRNAs

## Abstract

Choriocarcinoma stem-like cells (CSLCs) might be at the origin of choriocarcinoma development associated with drug resistance or relapse. Spalt-like transcription factor 4 (SALL4), which is considered to be a stemness-related gene, can be regulated by miRNAs. In this study, SALL4 result is associated with progression-free survival of choriocarcinoma patients and CSLC’s stemness characteristics. In addition, it could be downregulated by miR-497-5p by direct binding. miR-497-5p silencing by hypermethylation promoted malignant CSLC phenotype in vitro and in vivo. Furthermore, increased DNA methyltransferases (DNMTs) by SALL4 upregulation inhibited miR-497-5p expression via hypermethylation promotion. SALL4 appeared to be a key factor in promoting stemness phenotype of choriocarcinoma. Silencing miR-497-5p and SALL4 promotes choriocarcinoma progression and forms a feedback loop with DNMT-mediated epigenetic regulation, playing a crucial role in stemness maintenance in choriocarcinoma.

## Graphical abstract

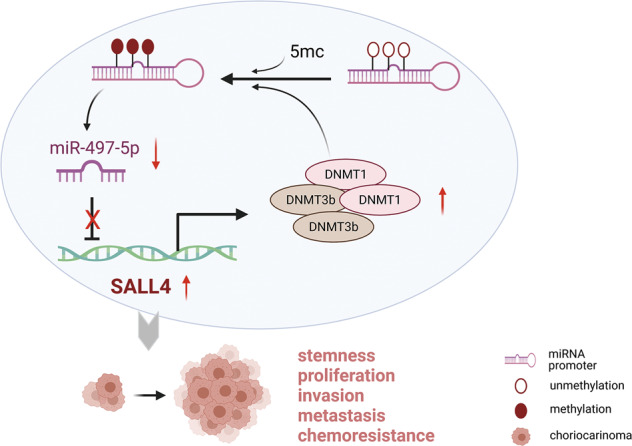

## Introduction

Choriocarcinoma is a gestational, highly malignant trophoblastic neoplasia, often arising after normal pregnancy, hydatidiform moles, or spontaneous abortions [1]. Chemotherapy is the first-line treatment of choriocarcinoma, however, 15–25% of high‑risk choriocarcinoma develops resistance to chemotherapy or relapses following the completion of initial therapy [2]. Cancer stem cells are considered as involved in tumorigenesis [3], progression, and recurrence after therapy since they regulate long-term tumor growth and progression [4]. Our previous study isolated the choriocarcinoma stem-like cells (CSLCs) from the JEG-3 cells, the most typical cell model of choriocarcinoma [5].

Spalt-like transcription factor 4 (SALL4) is a zinc-finger transcription factor detected in fetal gonadal germ cells [6]. Due to its oncogenic role, SALL4 has emerged as a tumor marker in many tumors [7], especially germ cell tumors [8, 9]. Aberrant SALL4 expression results in cell proliferation promotion in choriocarcinoma [10], and SALL4 is a useful biomarker to distinguish it from other trophoblastic tumors [11].

Stem-like phenotype in cancer is the result of genetic and epigenetic alterations leading to the expression of specific genes. The role of epigenetic modifications, such as DNA methylation and non-coding RNAs, has emerged in driving tumorigenesis [12]. MicroRNAs (miRNAs) are small non-coding RNAs, binding to mRNA’s 3′UTRs to suppress gene transcription and protein translation. Many miRNAs participate in tumor formation and development as oncogenes or tumor suppressors, via regulating target genes [13, 14]. DNA methylation, which has been widely reported to downregulate the target mRNA expression in malignant tumor phenotypes [15, 16], is controlled by DNA methyltransferases (DNMTs) via catalyzing 5-mC formation in the CpG islands [17]. The epigenetic modifications mediated by DNMTs in human trophoblastic cells regulate migration and invasion [18]. CpG island hypermethylation in miRNAs is one of the most common mechanisms to attenuate miRNA stimulatory or inhibitory effects on tumorigenesis [19, 20]. Some tumor-suppressive miRNAs, such as miR-144/451a and miR-152/148b, are silenced as a consequence of their promoter regions’ hypermethylation, leading to tumor growth enhancement in cancers [21, 22].

In the present work, we identified miR-497-5p and its target gene SALL4 as crucial factors in regulating stemness characteristics and malignant phenotype of choriocarcinoma. Mechanistically, miR-497-5p targets SALL4 to enhance DNMTs, which in turn inhibits miR-497-5p via hypermethylation promotion.

## Results

### SALL4 overexpression is correlated with choriocarcinoma prognosis

**Fig. 1:**
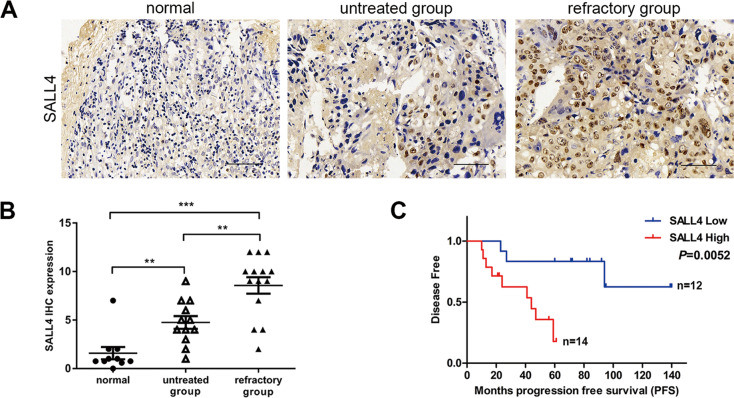
SALL4 overexpression is correlated with choriocarcinoma prognosis. **A** SALL4 protein levels in 10 normal and 26 choriocarcinoma tissues by using IHC (magnifications: ×400). **B** SALL4 expression in 10 normal and 26 choriocarcinoma tissues according IHC scores. **C** Progression-free survival time between low-SALL4-expression group and high-SALL4-expression group in choriocarcinoma tissues. Data represent mean ± SD from at least three independent experiments. **P < 0.01, ***P < 0.001.

Choriocarcinoma with high risk showed a higher SALL4 expression than with low risk. Furthermore, the different levels of SALL4 expression correlated with FIGO stages (Table 1, *P* < 0.05). SALL4 expression is the independent risk factor correlated with the PFS of choriocarcinoma (Table 2, HR = 7.045, *P* = 0.016).To evaluate the role of SALL4 in choriocarcinoma, SALL4 protein expression was analyzed in normal, untreated, and refractory groups by immunohistochemistry (IHC). Refractory group showed a higher SALL4 expression than untreated group (Fig. 1A, B, *P* < 0.01). Furthermore, we divided the choriocarcinoma samples into two groups based on the mean value (6.80) of SALL4 expression. Patients with tumors that expressed SALL4 at high levels had lower progression-free survival (PFS) when compared to patients with tumors with low SALL4 expression (Fig. 1C, *P* = 0.0052).

**Table 1:** SALL4 expression and clinicopathological features in 26 choriocarcinoma patients.

Clinical characteristic	Number of patients	SALL4		*P* value
		Low expression	High expression	
Age (year)				
<35	10	5	5	0.756
≥35	16	7	9	
Risk scores				
≤6	11	9	2	0.002
>6	15	3	12	
FIGO stages				
I	5	5	0	<0.001
II	8	6	2	
III–IV	13	1	12	
Chemotherapy				
No	12	9	3	0.006
Yes	14	3	11

**Table 2:** Cox regression of SALL4 for progression-free survival (PFS) in choriocarcinoma.

Variable	PFS	
	HR (95% CI)	*P* value
Age (year)		
<35 vs. ≥35	1.128 (0.986–1.290)	0.079
Risk scores		
≤6 vs. >6	1.927 (1.345–2.760)	<0.001
FIGO stages		
I–II vs. III–IV	4.296 (1.144–16.135)	0.031
SALL4 expression		
Low vs. high	7.045 (1.470–33.763)	0.015

### SALL4 enhances choriocarcinoma CSLC’s malignant phenotype

**Fig. 2:**
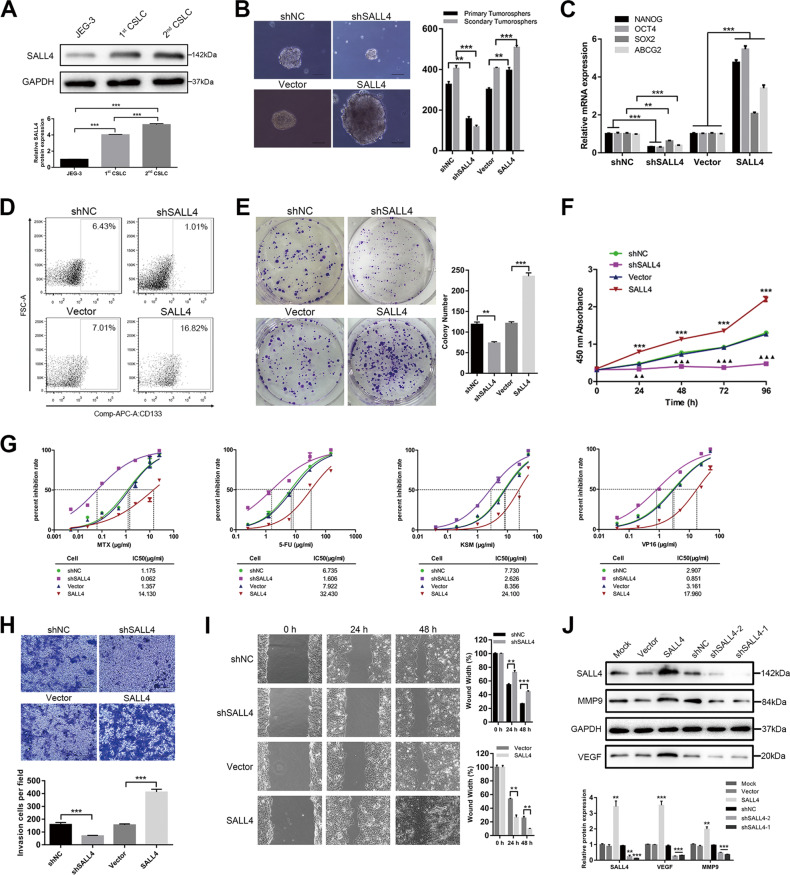
SALL4 enhances choriocarcinoma CSLC’s malignant phenotype. **A** Western blot analysis of SALL4 protein levels in JEG-3 cells versus CSLCs’ generations (top) and relative expression compared with JEG-3 cells (bottom). **B** Representative pictures (left, magnifications: ×100) of spheres and counts (right) of two generations. **C** Stemness gene expression by qRT-PCR analysis. **D** Flow cytometry plots of CD133 cell-surface protein expression. **E** Colony-formation assays using single CSLCs (left) and colony number counts (right). **F** OD values of shSALL4 or SALL4 cells compared with control cells on proliferation ability for 96 h by CCK-8 assay (*SALL4 vs. vector; ^▴^shSALL4 vs. shNC). **G** IC_50_ dose response curves of methotrexate, fluorouracil, dactinomycin, and etoposide. **H** Representative images (top, magnifications: ×100) and quantification (bottom) of invaded cells using transwell assay. **I** Migration images (left, magnifications: ×100) and quantification (right) of width area by wound healing assay. **J** Effects of SALL4 overexpression or silencing on VEGF and MMP-9 protein by western blotting. Data represent mean ± SD from at least three independent experiments. **P < 0.01, ***P < 0.001.

Based on the serum-free suspension culture, we obtained two choriocarcinoma CSLC generations from JEG-3 cells and we found that SALL4 protein expression increased as CSLCs’ generation increase (Fig. 2A, *P* < 0.001). To investigate the role of SALL4 in CSLCs, we established two SALL4-silencing cell lines (shSALL4-1, referred to as shSALL4. shSALL4-2 showed in Supplementary Fig. 1) and a SALL4-overexpressing cell line (SALL4 cells), and we also transfect cells with the corresponding controls (shNC, vector cells), we enriched the two different CSLCs and compared their malignant phenotypes. As shown in Fig. 2B, greater and more spheres were obtained in SALL4 cells, whereas the spheres formed by shSALL4 cells were smaller and less in number than control cells. In terms of stem-cell markers, SALL4 cells displayed higher pluripotency-associated gene expression (Fig. 2C, *P* < 0.01) and CD133 cell-surface protein expression (Fig. 2D, *P* < 0.05). At a functional level, SALL4 overexpression significantly enhanced colony formation and proliferation ability of CSLCs (Fig. 2E, F, *P* < 0.05). SALL4 cells showed more resistance to chemotherapy including MTX, 5-FU, KSM, and VP-16 (Fig. 2G, *P* < 0.05). Furthermore, SALL4 overexpression enhanced CSLCs’ invasion and migration ability (Fig. 2H–J, *P* < 0.01). In contrast, SALL4 silencing displayed a reduced ability of colony formation, proliferation, drug resistance, invasion, and migration in vitro. These results suggested a role of SALL4 as a tumor promoter in choriocarcinoma CSLCs.

### miR-497-5p regulates SALL4 directly in choriocarcinoma CSLCs

**Fig. 3:**
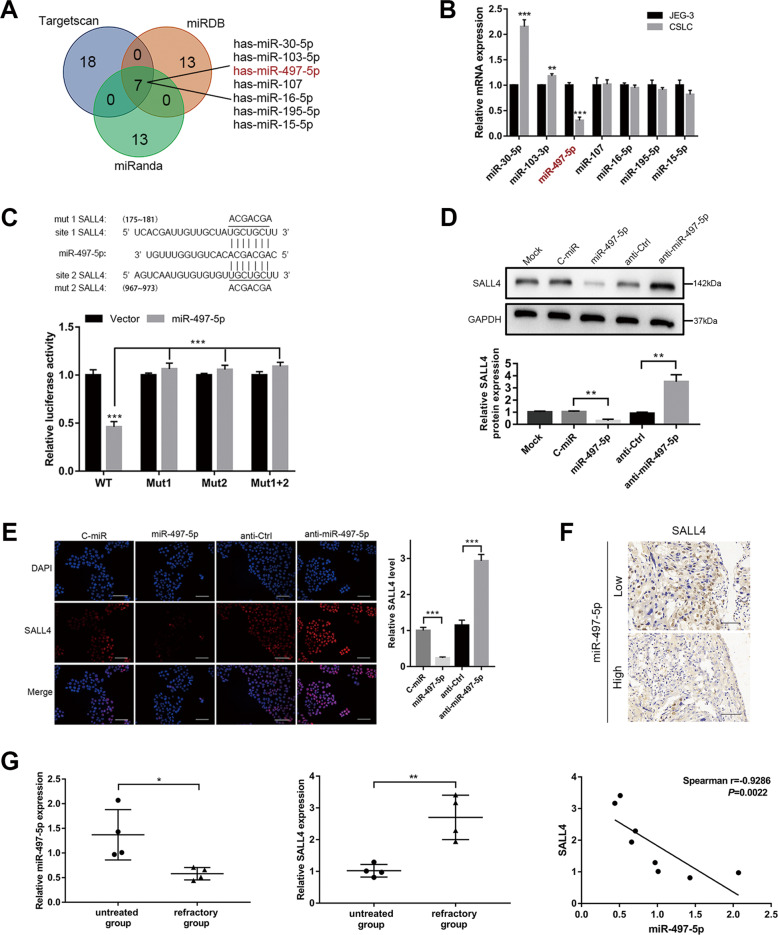
miR-497-5p regulates SALL4 directly in choriocarcinoma CSLCs. **A** TargetScan, miRDB, and miRanda tools’ analysis of miRNAs that potentially target SALL4. **B** qRT-PCR analysis of 7 miRNA levels between JEG-3 and CSLCs. **C** Schematic representation of SALL4 3′UTR showed two putative miR-497-5p binding sites, including the wild-type and mutant (top); 293T cells were transfected with indicated expression plasmids. Luciferase activities were measured (bottom). **D** Western blot analysis of SALL4 protein levels (top) and quantification (bottom). **E** SALL4 localized in the nucleus by immunofluorescent analysis (left, magnifications: ×200) and quantification (right). **F** Representative images of SALL4 expression detected by IHC (magnifications: ×400). **G** qRT-PCR analysis showing that miR-497-5p inversely associated with SALL4 in choriocarcinoma tissues. Data represent mean ± SD from at least three independent experiments. **P < 0.01, ***P < 0.001.

To explore the SALL4 mechanism of action in CSLCs, we focused on miRNAs that might target SALL4. Three miRNA prediction tools, TargetScan, miRDB, and miRanda, were used to search for potential miRNAs. The predicted target sites of seven miRNAs (Fig. 3A) were considered to be best-supported based on the criteria (Supplementary material 1). miR-497-5p was selected based on the miRNA’s expression comparison between JEG-3 and CSLCs. Only miR-497-5p expression significantly decreased in CSLCs (Fig. 3B, *P* < 0.001). After sequence alignment, two binding sites of miR-497-5p in the 3′UTR of SALL4 were identified (Fig. 3C). Luciferase reporter assay was used to test if SALL4 was a direct target of miR-497-5p. As expected, luciferase assay results showed that miR-497-5p overexpression markedly decreased luciferase activity in the 3′UTR of wild-type SALL4, but failed to suppress luciferase activity in the 3′UTR mutant, without any difference between site 1 mutant and site 2 or both (Fig. 3C, *P* < 0.001). Western blot analyses (Fig. 3D, *P* < 0.01) were performed, showing that miR-497-5p overexpression reduced SALL4 levels, whereas, miR-497-5p silencing abrogated the inhibition of SALL4. SALL4 immunofluorescence analysis confirmed the repression of SALL4 expression by miR-47-5p overexpression in choriocarcinoma cells (Fig. 3E, *P* < 0.001). All these results further indicated that SALL4 was directly and negatively regulated by miR-497-5p.

We divided the choriocarcinoma samples into two groups based on the median value (0.97) of miR-497-5p expression. SALL4 protein levels were higher in low-miR-497-5p-expression samples than in high-miR-497-5p-expression samples (Fig. 3F, *P* < 0.05). Moreover, in the eight fresh choriocarcinoma tissues, the refractory group showed low miR-497-5p and high SALL4 expressions by qRT-PCR, whereas the untreated group showed the opposite result (Fig. 3G). Overall, these results suggested that miR-497-5p expression was negatively correlated with SALL4 expression (Fig. 3G, Spearman *r* = -0.9286, *P* = 0.0022). Furthermore, silencing miR-497-5p promotes stem-like characteristics in choriocarcinoma CSLCs (Supplementary Results, Supplementary Fig. 2).

### Effects of miR-497-5p and SALL4 on choriocarcinoma in vivo

**Fig. 4:**
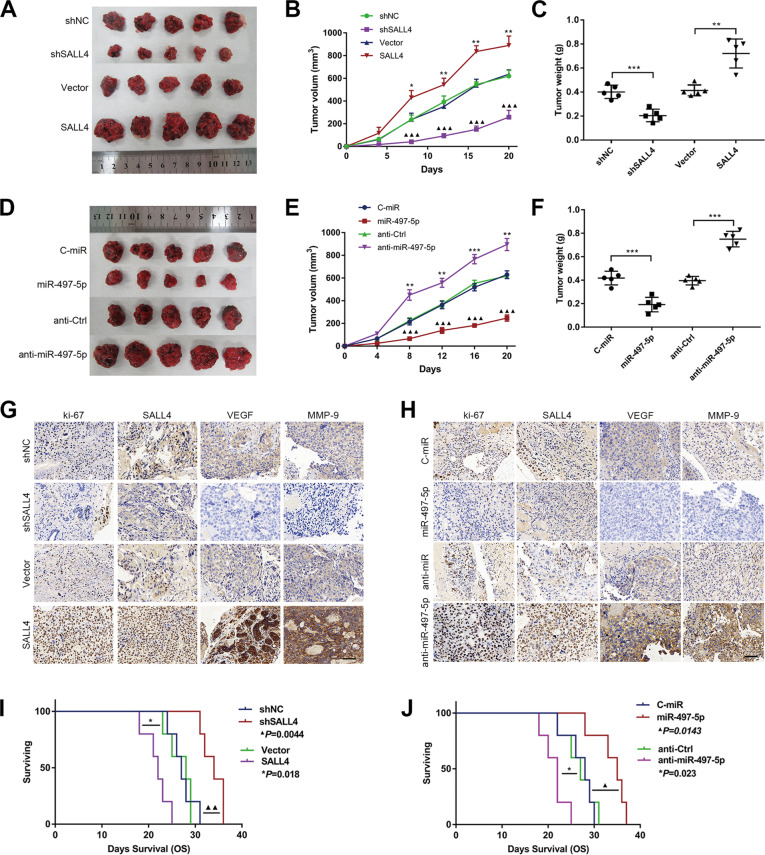
Effects of miR-497-5p and SALL4 on choriocarcinoma in vivo. **A** Growth of subcutaneous xenograft tumors for following groups of shNC, shSALL4, vector, or SALL4, with 5 tumors per group. **B** Tumor volumes were measured every 4 days for 3 weeks (*SALL4 vs. vector; ^▲^shSALL4 vs. shNC). **C** Tumor weight in the indicated mice. **D** Comparison of tumor sizes among C-miR, miR-497-5p, anti-Ctrl, or anti-miR-497-5p groups (*n* = 5 each group). **E** Tumor volumes were measured every 4 days for 3 weeks (*anti-miR vs. anti-Ctrl; ^▲^miR vs. C-miR). **F** Tumor weight in the indicated mice. **G** IHC staining displaying the Ki-67, SALL4, VEGF, and MMP-9 protein expression among shNC, shSALL4, vector, or SALL4 groups (IHC, magnifications: ×400). **H** IHC staining displaying the Ki-67, SALL4, VEGF, and MMP-9 protein expression among C-miR, miR-497-5p, anti-Ctrl, or anti-miR-497-5p groups (IHC, magnifications: ×400). **I** Kaplan-Meier curves of shSALL4 and SALL4 mice, compared with the control. **J** Kaplan-Meier curves of miR-497-5p and anti-miR-497-5p mice, compared with the control. Data represent mean ± SD from at least three independent experiments. *P < 0.05, **P < 0.01, ***P < 0.001.

We further assessed the effects of SALL4 and miR-497-5p on tumor progression in vivo. As shown in Fig. 4A–F, consistently with our in vitro results, SALL4 overexpression and miR-497-5p silencing promoted tumor xenograft’s growth. After 3 weeks, SALL4 cell- and anti-miR-497-5p cell-injected mice showed a significantly higher tumor volume and weight than control groups. In contrast, shSALL4 and miR-497-5p groups showed lower tumor volume and weight. Furthermore, IHC analysis showed that SALL4- and anti-miR-497-5p-treated xenografts displayed elevated percentages of Ki-67-positive cells and increased VEGF and MMP-9 expression (Fig. 4G, H). In addition, Kaplan-Meier analysis revealed that mice implanted with SALL4 and anti-miR-497-5p cells survived significantly less, while mice implanted with shSALL4 and miR-497-5p cells survived more than control groups (Fig. 4I, J, *P* < 0.05). Taken together, these results strengthen the hypothesis that SALL4 overexpression and miR-497-5p silencing promoted choriocarcinoma progression also in vivo.

### DNA hypermethylation causes miR-497-5p silencing and suppresses choriocarcinoma CSLCs

**Fig. 5:**
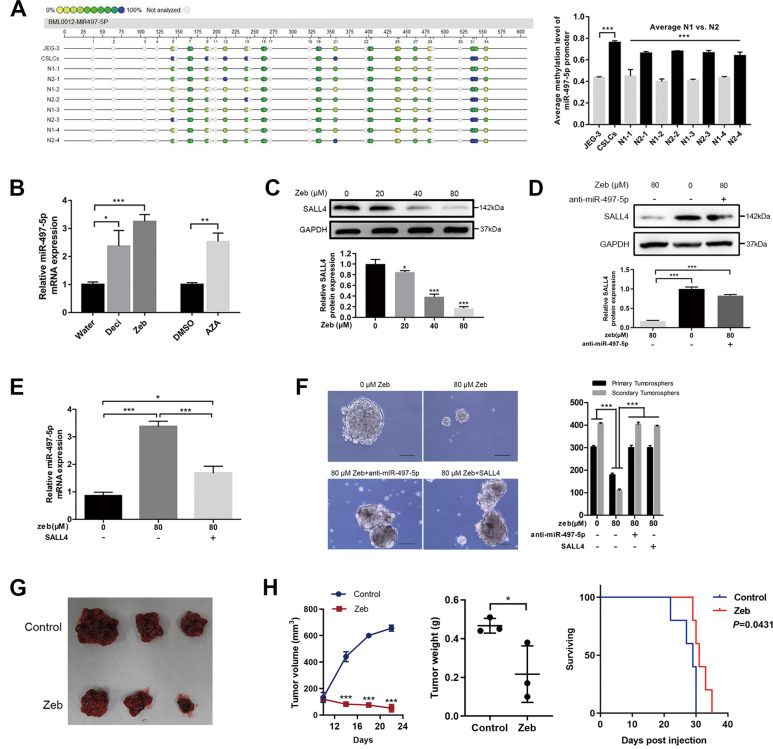
DNA hypermethylation causes miR-497-5p silencing and suppresses choriocarcinoma CSLCs. **A** Profiling of methylation levels of CpG sites in the miR-497-5p upstream region is presented as an epigram (left) and average methylation level of miR-497-5p promoter between the indicated groups (right). **B** qRT-PCR demonstrated miR-497-5p expression in CSLCs after treatment with decitabine, zebularine, or 5-zazcytidine for 7 days compared with control groups. **C** Western blot analysis of SALL4 protein levels (up) and quantification (bottom) with zebularine treatment. **D** Western blot analysis of SALL4 protein levels (up) and quantification (bottom) with zebularine treatment and anti-miR-497-5p. **E** qRT-PCR demonstrated miR-497-5p expression in CSLCs after treatment with 80 μM zebularine and overexpressed SALL4 compared with control groups. **F** Representative images of sphere (left, magnifications: ×100) and sphere counts (right) with different groups. **G** Xenograft tumor in nude mice treated with zebularine at 200 mg/kg/day for 10 days. **H** Tumor volumes were measured every 4 days for 3 weeks (left). Tumor weight in the mice with zebularine treatment (middle). Survival curve of tumor-bearing mice between the zebularine group and control group (right). Data represent mean ± SD from at least three independent experiments. *P < 0.05, **P < 0.01, ***P < 0.001.

Dysregulation of epigenetic mechanisms is an important cause of abnormal miRNA expression in cancers [23]. In ovarian cancer and breast cancer, miR-497 downregulation is due to DNA methylation [24, 25]. Hence, we first analyzed the genomic DNA sequence within miR-497-5p promoter regions and found the presence of CpG islands. Next, Sequenom EpiTYPER MassArray was used to assess the DNA methylation level of 21 CpG sites upstream of the miR-497-5p in JEG-3 cells and CSLCs, or in four untreated samples (N1-1 to N1-4) and four refractory samples (N2-1 to N2-4). The results showed that the average methylation in all CpG sites of miR-497-5p was significantly higher in CSLCs and refractory group (Fig. 5A, *P* < 0.001). Three DNMT inhibitors were used to test the effect on miR-497-5p expression level. Decitabine, zebularine, and 5-zazcytidine significantly reversed miR-497-5p expression (2.36-, 3.25-, and 2.53-fold, respectively), but the effect of zebularine, which is stable and preferentially targets cancer cells without toxicity in normal cells and mice [26], was prominent (Fig. 5B, *P* < 0.05). Zebularine treatment during sphere formation at four different doses remarkably altered the level of SALL4 protein in CSLCs in a dose-dependent manner (Fig. 5C, *P* < 0.05). The results indicate that DNA hypermethylation indeed repressed miR-497-5p expression. To confirm that the inhibitory effect of zebularine on SALL4 was mainly achieved by changing the methylation state of miR-497-5p, we added the rescue experiment. Here, 80 μM zebularine can greatly reduce the expression of SALL4, but SALL4 cannot be reduced by 80 μM zebularine remarkably in the choriocarcinoma cells which interfered by anti-miR-497-5p treatment (Fig. 5D, *P* < 0.001). Zebularine, as the inhibitor of DNMTs, can increase miR-497-5p expression by inhibiting DNA methylation (Fig. 5B). However, as shown in Fig. 5E, the expression of miR-497-5p was increased by 80 μM zebularine significantly, but rescued by the overexpressed SALL4 in choriocarcinoma cells (Fig. 5E, *P* < 0.001). Moreover, from the cell sphere formation, it can be intuitively seen that the tumor-suppressive effect of zebularine can be rescued by anti-miR-497-5p and overexpressed SALL4 (Fig. 5F, *P* < 0.001). Furthermore, in vivo, choriocarcinoma CSLC’s tumor-bearing nude mice were subjected to an oral treatment with zebularine (200 mg/kg) every day for 10 days until tumor growth reached 100 mm^3^. Two weeks after the last treatment, mice were sacrificed and the tumor was removed and observed (Fig. 5G). Tumors displayed a significant shrinkage after zebularine treatment, both in tumor volume and weight, and the mice exhibited longer survival (Fig. 5H, *P* < 0.05). Overall, these data showed that methyltransferase activity inhibition can overcome the stemness potential of choriocarcinoma CSLCs.

### DNMTs regulate miR-407-5p-SALL4 network

**Fig. 6:**
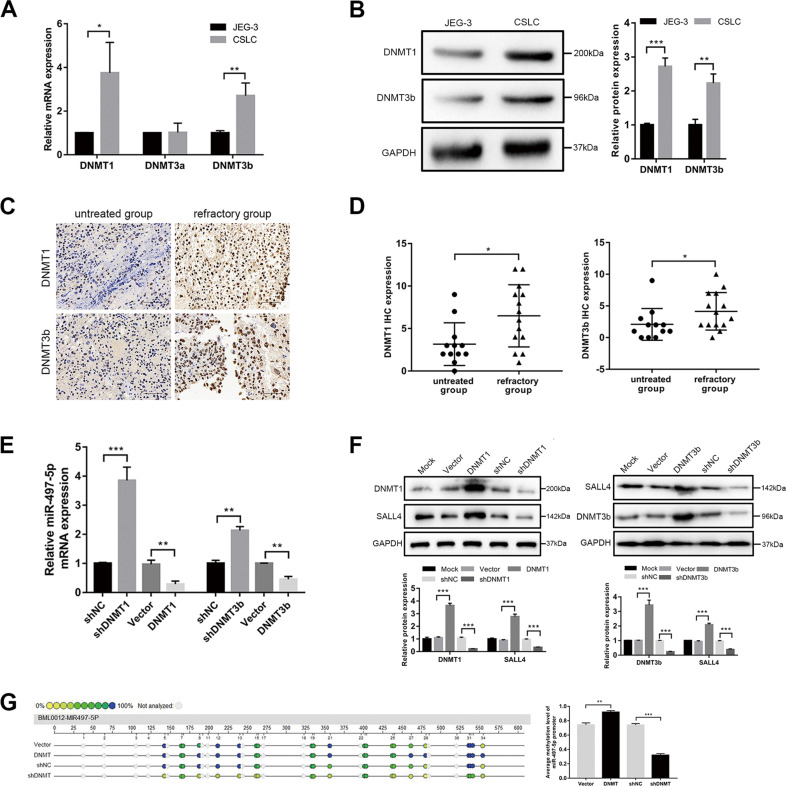
DNMTs regulate miR-497-5p/SALL4 axis. **A** qRT-PCR assay for DNMT1, DNMT3a, and DNMT3b levels in JEG-3 cells and CSLCs. **B** Western blot analysis for DNMT1 and DNMT3b in CSLCs compared with JEG-3 cells. **C** IHC staining of DNMT1 and DNMT3b in 26 choriocarcinoma tissues (magnifications: ×400). **D** DNMT1 and DNMT3b expression in 14 refractory group compared with 12 untreated group according to IHC scores. **E** qRT-PCR analysis for miR-497-5p levels in shDNMT1/3b and DNMT1/3b groups compared with control groups in CSLCs. **F** Respective images (top) of SALL4 levels in shDNMT1/3b and DNMT1/3b groups compared with control groups in CSLCs by western blot analysis and quantification (bottom). **G** Profiling of methylation levels of CpG sites in the miR-497-5p upstream region are presented as an epigram (left) and quantification by average methylation levels (right). Data represent mean ± SD from at least three independent experiments. *P < 0.05, **P < 0.01, ***P < 0.001.

We previously observed the effect of miR-497-5p promoter hypermethylation and the opposite effect of DNMT inhibitors on choriocarcinoma CSLCs. To test whether DNMTs contribute to miR-497-5p downregulation, we evaluated DNMT expression in CSLCs and choriocarcinoma tissues. Our results showed that DNMT1 and DNMT3b were highly expressed in CSLCs compared with JEG-3 cells (Fig. 6A, B, *P* < 0.05). In the 14 refractory-group tissues, high levels of DNMT1 and DNMT3b were found compared to the low level in the 12 untreated-group tissues (Fig. 6C, D, *P* < 0.05). Similar to methyltransferase inhibitor, DNMT1/3b knockdown specifically increased miR-497-5p transcription (Fig. 6E, *P* < 0.01). Furthermore, SALL4 expression that is actually related to miR-497-5p was reduced as a consequence of DNMT1/3b knockdown and increased by DNMT1/3b overexpression (Fig. 6F, *P* < 0.001). However, the effect of DNMTs can be rescued partially by overexpressed miR-497-5p. It means that DNMTs can promote SALL4 expression, at least most of them by changing the methylation state of miR-497-5p (as shown in Supplementary Fig. 3). We failed to find DNMT binding sites in miR-497-5p promoter, thus, we tested whether miR-497-5p was regulated by DNMTs via hypermethylation repression. As Fig. 6G shows, DNMT1/3b overexpression promoted hypermethylation of miR-497-5p in CSLCs, measured by Sequenom MassARRAY (*P* < 0.01). Taken together, these results suggested that DNMTs regulated miR-497-5p-SALL4 network via hypermethylation repression.

### SALL4 regulates DNMTs, with miR-497-5p forming a feedback loop

**Fig. 7:**
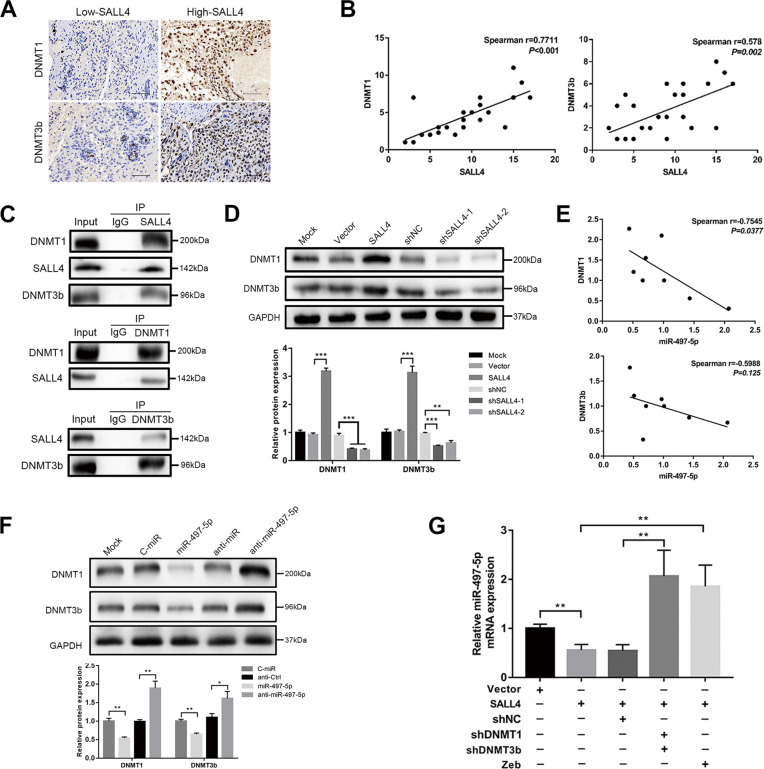
SALL4 regulates DNMTs, with miR-497-5p forming a feedback loop. **A** IHC staining of DNMT1 and DNMT3b in SALL4 low-expression group compared with high-expression group (IHC, magnifications: ×400). **B** Spearman correlation analysis for SALL4 and DNMT1 (left) or DNMT3b (right) based on IHC scores in 26 choriocarcinoma tissues. **C** Interactions between SALL4 and DNMT1 or DNMT3b in CSLCs. Co-IP assays showed endogenous SALL4 coprecipitated with DNMT1 and DNMT3b (top), and endogenous DNMT1 or DNMT3b coprecipitated with SALL4 (middle and bottom). **D** Respective images (top) and quantification (bottom) of DNMT1 and DNMT3b expression in CSLCs with SALL4 overexpression or silencing by western blotting. **E** Spearman correlation analysis for miR-497-5p and DNMT1 (top) or DNMT3b (bottom) based on qRT-PCR data of 8 choriocarcinoma tissues. **F** Respective images (top) and quantification (bottom) of DNMT1 and DNMT3b protein levels with miR-497-5p overexpression or silencing by western blot analysis. **G** qRT-PCR analysis for miR-497-5p with co-transfection of SALL4, shDNMT1, and shDNMT3b in indicated groups. Data represent mean ± SD from at least three independent experiments. *P < 0.05, **P < 0.01, ***P < 0.001.

SALL4 has been reported to suppress transcription through recruitment of DNMTs, including DNMT1, DNMT3a, and DNMT3b [27]. Because of DNMTs’ effect on regulating miR-497-5p, we wondered whether SALL4 regulates DNMTs in choriocarcinoma CSLCs. In choriocarcinoma tissues, samples with high SALL4 expression also expressed higher DNMT1 and DNMT3b levels compared to samples with low SALL4 expression (Fig. 7A). Subsequently, we performed DNMT1, DNMT3b, and SALL4 expression correlation analysis, confirming a significant positive correlation among patient samples (Fig. 7B, *P* < 0.01). To further study if SALL4 could actively recruit DNMT proteins, endogenous Co-IP followed by western blotting analysis was used to demonstrate that SALL4 successfully interacted with DNMT1 and DNMT3b (Fig. 7C). Moreover, SALL4 overexpression significantly promoted DNMT1 and DNMT3b expression in CSLCs (Fig. 7D, *P* < 0.01). On the basis of all the above results, we hypothesized whether a feedback loop exists in the progression of CSLCs. First, in eight choriocarcinoma tissues, as shown in Fig. 7E, miR-497-5p transcript level was remarkably negatively correlated with DNMT1 (Spearman *r* = −0.7545, *P* = 0.0377) but not with DNMT3b (Spearman *r* = −0.5988, *P* = 0.125). Furthermore, in CSLCs, miR-497-5p silencing increased DNMT1 and DNMT3b protein expression, while miR-497-5p overexpression reduced them (Fig. 7F, *P* < 0.05). To obtain a further confirmation, co-transfection of SALL4, shDNMT1, and shDNMT3b was performed to enrich CSLCs. SALL4 overexpression reduced miR-497-5p expression, but importantly, shDNMTs significantly abrogated the inhibition. Consistently, zebularine also reversed SALL4 inhibition on miR-497-5p (Fig. 7G, *P* < 0.01).

Overall, our study strongly supports the role of SALL4 and miR-497-5p in choriocarcinoma stemness progression and illustrates the existence of a feedback loop DNMTs/miR-497-5p/SALL4 in choriocarcinoma CSLCs.

## Discussion

In this study, we demonstrated the role of SALL4 in the progression of choriocarcinoma CSLCs, being directly regulated by miR-497-5p, playing pivotal roles in stemness and malignant phenotype. In addition, we identified a DNMTs/miR-497-5p/SALL4 feedback loop as an automatic driver in the modulation of choriocarcinoma stem-like growth.

SALL4 is known to be abnormally expressed in multiple tumors, where it plays important roles in malignant processes, such as drug resistance, cell survival, and stemness [28]. Recent studies show that gestational trophoblastic neoplasia, especially choriocarcinoma, displays high-SALL4-expression level [10, 11], which can be explained by its important functions in embryonic stem cell maintenance [29]. Consistently, in this study SALL4 expression was analyzed in 36 cases, being upregulated in 26 choriocarcinoma tissues and gradually increasing in the choriocarcinoma with high-risk or unremission after chemotherapy. Due to the difficulty in obtaining choriocarcinoma clinical specimens with strictly according to FIGO staging, we cannot evaluate the correlation between tumor staging and SALL4 exactly. However, we identified that high SALL4 level was associated with poor choriocarcinoma prognosis. Malignant choriocarcinoma is insensitive to chemotherapeutics with stemness characteristics. Human chorionic gonadotrophin (hCG) is the main gestational trophoblastic neoplasia biomarker. However, it cannot be used to predict the outcome of therapies. New prognostic indicators should be selected to evaluate choriocarcinoma outcome for a suitable combination chemotherapy by the selection of high-risk patients. Our data suggested that SALL4 might be a candidate deserving further study.

Numerous studies reported that miR-497-5p is closely related to various carcinomas as a tumor suppressor [30, 31]. As the direct suppressor of SALL4, our results showed that miR-497-5p silencing significantly increased, but miR-497-5p overexpression inhibited SALL4 in CSLCs. In order to inspect if miR-497-5p downregulation was functionally involved in choriocarcinoma CSLCs, functional experiments were carried out both in vitro and in vivo, indicating a tumor suppressor role of miR-497-5p in choriocarcinoma CSLCs. Promoter hypermethylation of multiple genes in choriocarcinoma [32], together with increasing evidences support the idea that some important miRNAs’ expression is influenced by epigenetic modifications, such as DNA methylation [33, 34]. In ovarian cancer and breast cancer, miR-497 was downregulated by DNA methylation, which served as a tumor suppressor and a diagnostic marker [24, 25]. Accordingly, promoter aberrant methylation suppressed miR-497-5p expression in both choriocarcinoma CSLCs and tissues. Besides, DNMT1 and DNMT3b were negatively correlated with miR-497-5p in eight choriocarcinoma tissues. DNMT1 maintains an established DNA methylation pattern, whereas DNMT3a and DNMT3b set up DNA methylation patterns during early development. As key enzymes, DNMTs were recognized as antitumor drug targets in terms of epigenetic therapy [35]. In choriocarcinoma CSLCs, miR-497-5p expression was downregulated by DNMT-mediated hypermethylation regulation.

In the formation and progression of choriocarcinoma CSLCs, only a constant driving factor exists, the “stemness” of tumors can be immortalized. Hence, the positive feedback loop DNMTs/miR-497-5p/SALL4 was proposed in this study. Positive feedback loops are normally seen in regulatory mechanisms, resulting in a silent developmental action in a dynamic background pathway, but triggering a robust response upon real stimuli at the right time and place [36]. SALL4, as a transcription factor, can recruit DNMTs. Redundant DNMTs promote miR-497-5p promoter methylation, resulting in miR-497-5p silencing. Thus, in turn, SALL4 transcription is enhanced due to the removal of the suppression on SALL4 by silencing miR-497-5p. It is tempting to speculate that, once induced by any aberrance, the DNMTs/miR-497-5p/SALL4 feedback loop allows CSLCs to become more autonomous, such as drugs administered in an insufficient dose, unsuitable treatments, or delayed efficient interventions can all trigger malignancy. This effect would enhance the ability of CSLCs to become more malignant or promote stemness, and also indicates that if any link is broken in the process, the feedback loop would turn around in the opposite direction, which hint the multi-target treatment in a vicious circle. The exact molecular function in the stemness maintenance might be context dependent, however, it needs further investigation due to our limited clinical specimens. A better understanding of the molecular mechanisms underlying stemness progression is necessary for developing successful strategies to treat malignant choriocarcinoma. Regardless, our identification of the remarkable role of SALL4 might suggest an exciting new approach for pharmacological intervention against choriocarcinoma.

## Materials and methods

### Ethics statement

All the investigations have been approved by the Ethics Committee Institute of Xiangya Hospital of Central South University (Changsha, Hunan, China).

### Patient information and tissue specimens

Patient characteristics are shown in Supplementary Table 1. Ten normal trophoblastic paraffin-embedded tissues of early-pregnancy patients with induced abortion and 26 choriocarcinoma tissues were obtained from the Pathology Department of Xiangya Hospital of Central South University between 2000 and 2019. According to the International Federation of Gynecology and Obstetrics (FIGO) risk prognostic factor scores (2002), a total score of 6 or less is considered as low risk [37]. Twelve low-risk samples without chemotherapy were collected and considered as untreated group, most of them obtained from uterine curettage to confirm the diagnosis. Fourteen samples with poor remission or tumor recurrence after multidrug therapy were obtained from surgery and considered as refractory group. Progression-free survival was defined as a time period from the initial surgical operation to tumor progression or recurrence. The last follow-up time was December 2020. Besides, eight fresh choriocarcinoma samples were obtained by surgical removal from Xiangya Hospital of Central South University between 2018 and 2020. Four samples for non-therapy choriocarcinoma with low-risk and four samples for refractory choriocarcinoma after multidrug chemotherapy were obtained and considered as untreated group and refractory group, respectively.

### Immunohistochmical (IHC) staining

Fresh tissues were fixed with formalin and paraffin embedded. All the tissue samples were cut into 4 μm sections. After deparaffinization and rehydration, sections were boiled in citrate buffer at 100 °C for 3 min and immersed in 3% H_2_O_2_ for 10 min for endogenous peroxidase blocking. First, the sections were incubated overnight with the antibody (Ki-67, 1:20, Millipore; SALL4, 1:500, Abcam; DNMT1, 1:200, CST; DNMT3b, 1:500, CST; VEGF, 1:500, CST; MMP-9, 1:300, CST). Then, washed with PBS and incubated in goat anti-rabbit IgG HRP (CST, Darmstadt, DE) for 30 min. Finally, stained with DAB (Solarbio, Beijing, China) and then analyzed the stained cells. Five fields were randomly selected. The protein IHC expression in tissues was normalized by IHC scores, according the method described previously [38].

### Cell culture

The human choriocarcinoma JEG-3 cell line, which was laboratory preserved, was purchased from the American Type Culture Collection (ATCC, Manassas, USA) [39]. Cells were cultured in DMEM high-glucose (Gibco, Grand Island, USA) supplemented with 10% FBS (Gibco, Grand Island, USA) at 37 °C in a 5% CO2 atmosphere.

### Sphere-formation assay

JEG-3 CSLCs were generated according the previous methods for 7 days [6]. The spheres were dissociated into single cells, re-cultured for another 7 days. The first-generation spheres were treated with decitabine (Deci, 0.5 μM), 5-zazcytidine (AZA, 75 μM), or Zebularine (Zeb, 80 μM) for 7 days. DNMT inhibitors were purchased from Selleck (Beijing, China).

### Real-time polymerase chain reaction (RT-PCR)

Total RNA was extracted using the TRIzol reagent (Life Technologies, Grand Island, USA) following the manufacturer’s instruction. qRT-PCR was performed using SYBR PremixEx TaqTM II (Life Technologies, Grand Island, USA) with ABI 7500 real-time PCR system (Thermo, MMAS, USA). Data were normalized to GAPDH levels and the expression level was calculated with 2^−△△Ct^ vs. control group. The primer sequences are listed in Supplementary Table 2.

### DNA extraction and methylation analyses

Genomic DNA was extracted from cells and eight choriocarcinoma tissues using the genomic DNA rapid extraction kit (TransGen Biotech, Beijing, China). An EZ DNA methylation kit (Zymo Research, Orange, CA) was used for treatment with sodium bisulfite of genomic DNA. The Sequenom MassARRAY platform (Biomiao Biological, Beijing, China) was used for the analysis of miR-497-5p methylation. Two pairs of primers were used, (aggaagagagTTTTTATATTTGGGGTGTAGGAGAA, caftaatacgactcactatagggagaaggctCCTAACCCCTAACCCTTTAAAAAAT) to amplify base pairs -29~582 upstream of miR-497-5p, (aggaagagagAAATTGTTTTGGAGATTTTAGAGGG, cagtaatacgactcactatagggagaaggctACACAAAAAACCTTAAATCCCTACC) to amplify base pairs 676−1242 around miR-497-5p. The spectra methylation ratios for each CpG site were generated by Epityper software version 1.0.

### Xenograft tumorigenicity assay

All animal care and experimental procedures were approved by the Institutional Animal Care and Use Committee of Central South University. BALB/c-nude mice (female, 4–5 weeks of age, 18–20 g) were purchased from the Center of Experimental Animal of Central South University. All the animals were randomly assigned. After 1 week of adaptation, the cells were inoculated subcutaneously into the left flank of mice. Tumor volume was calculated every 4 days using the equation (L × W^2^)/2. Mice were sacrificed 3 weeks after cell injection, and the tumors were stripped and weighed. For the overall survival analysis, whole survival time per mouse was recorded.

## Supplementary information

Supplementary Results.

Supplementary Figure 1.

Supplementary Figure 2.

Supplementary Figure 3.

Supplementary Figure legend.

Supplementary Tables.

Supplementary Materials and methods.

Supplementary material 1.

uncropped bolts.

Reproducibility Checklist form.

## References

[1] Froeling FE, Seckl MJ (2014). Gestational trophoblastic tumours: an update for 2014. Curr Oncol Rep.

[2] Powles T, Savage PM, Stebbing J, Short D, Young A, Bower M (2017). A comparison of patients with relapsed and chemo‑refractory gestational trophoblastic neoplasia. Br J Cancer.

[3] Visvader JE (2011). Cells of origin in cancer. Nature.

[4] Gupta PB, Onder TT, Jiang G, Tao K, Kuperwasser C, Weinberg RA (2019). Identification of selective inhibitors of cancer stem cells by high-throughput screening. Cell.

[5] Cai J, Peng T, Wang J, Zhang J, Hu H, Tang D (2019). Isolation, culture and identification of choriocarcinoma stem-like cells from the human choriocarcinoma cell-line JEG-3. Cell Physiol Biochem.

[6] Kong NR, Bassal MA, Tan HK, Kurland JV, Yong KJ, Young JJ (2021). Zinc finger protein SALL4 functions through an AT-rich motif to regulate gene expression. Cell Rep.

[7] Sun J, Tang Q, Gao Y, Zhang W, Zhao Z, Yang F (2020). VHL mutation-mediated SALL4 overexpression promotes tumorigenesis and vascularization of clear cell renal cell carcinoma via Akt/GSK-3β signaling. J Exp Clin Cancer Res.

[8] Cao D, Humphrey PA, Allan RW (2009). SALL4 is a novel sensitive and specific marker for metastatic germ cell tumors, with particular utility in detection of metastatic yolk sac tumors. Cancer.

[9] Al-Obaidy KI, Williamson SR, Shelman N, Idrees MT, Ulbright TM (2021). Hepatoid teratoma, hepatoid yolk sac tumor, and hepatocellular carcinoma: a morphologic and immunohistochemical study of 30 cases. Am J Surg Pathol.

[10] Zhao H, Wu L, Wu J, Yu H, Zhou J, Luan B (2018). Aberrantly expressed SALL4 promotes cell proliferation via β-catenin/c-Myc pathway in human choriocarcinoma cells. Reprod Sci.

[11] Stichelbout M, Devisme L, Franquet-Ansart H, Massardier J, Vinatier D, Renaud F (2016). SALL4 expression in gestational trophoblastic tumors: a useful tool to distinguish choriocarcinoma from placental site trophoblastic tumor and epithelioid trophoblastic tumor. Hum Pathol.

[12] Feinberg AP, Ohlsson R, Henikoff S (2006). The epigenetic progenitor origin of human cancer. Nat Rev Genet.

[13] Esquela-Kerscher A, Slack FJ (2006). Oncomirs-microRNAs with a role in cancer. Nat Rev Cancer.

[14] Li X, Wu P, Tang Y, Fan Y, Liu Y, Fang X (2020). Down-regulation of MiR-181c-5p promotes epithelial-to-mesenchymal transition in laryngeal squamous cell carcinoma via targeting SERPINE1. Front Oncol.

[15] Vural S, Palmisano A, Reinhold WC, Pommier Y, Teicher BA, Krushkal J (2021). Association of expression of epigenetic molecular factors with DNA methylation and sensitivity to chemotherapeutic agents in cancer cell lines. Clin Epigenetics.

[16] Lee M, Nam HY, Kang HB, Lee WH, Lee GH, Sung GJ (2021). Epigenetic regulation of p62/SQSTM1 overcomes the radioresistance of head and neck cancer cells via autophagy-dependent senescence induction. Cell Death Dis.

[17] Robertson KD (2001). DNA methylation, methyltransferases, and cancer. Oncogene.

[18] Rahnama F, Shafiei F, Gluckman PD, Mitchell MD, Lobie PE (2006). Epigenetic regulation of human trophoblastic cell migration and invasion. Endocrinology.

[19] Barter MJ, Bui C, Young DA (2012). Epigenetic mechanisms in cartilage and osteoarthritis: DNA methylation, histone modifications and microRNAs. Osteoarthr Cartil.

[20] Chuang JC, Jones PA (2007). Epigenetics and microRNAs. Pediatr Res.

[21] Zhao J, Li H, Zhao S, Wang E, Zhu J, Feng D (2021). Epigenetic silencing of miR-144/451a cluster contributes to HCC progression via paracrine HGF/MIF-mediated TAM remodeling. Mol Cancer.

[22] Khajehnoori Sahel, Zarei Fatemeh, Mazaheri Mahta, Dehghani-Firoozabadi Ali (2020). Epidrug modulated expression of MiR-152 and MiR-148a reverse cisplatin resistance in ovarian cancer cells: an experimental in-vitro study. Iran J Pharm Res.

[23] Grimson A, Farh KK, Johnston WK, Garrett-Engele P, Lim LP, Bartel DP (2007). MicroRNA targeting specificity in mammals: determinants beyond seed pairing. Mol Cell.

[24] Xu S, Fu GB, Tao Z, OuYang J, Kong F, Jiang BH (2015). MiR-497 decreases cisplatin resistance in ovarian cancer cells by targeting mTOR/P70S6K1. Oncotarget.

[25] Tao S, Li H, Ma X, Lian B, He J, Gao Y (2020). Methylation-mediated silencing of microRNA-497 promotes breast cancer progression through up-regulation of Mucin1. Front Oncol.

[26] Cheng JC, Yoo CB, Weisenberger DJ, Chuang J, Wozniak C, Liang G (2004). Preferential response of cancer cells to zebularine. Cancer Cell.

[27] Misawa K, Misawa Y, Mima M, Yamada S, Imai A, Mochizuki D (2020). Overexpression of Sal-like protein 4 in head and neck cancer: epigenetic effects and clinical correlations. Cell Oncol.

[28] Tatetsu H, Kong NR, Chong G, Amabile G, Tenen DG, Chai L (2016). SALL4, the missing link between stem cells, development and cancer. Gene.

[29] Yang J, Chai L, Fowles TC, Alipio Z, Xu D, Fink LM (2008). Genome-wide analysis reveals SALL4 to be a major regulator of pluripotency in murine-embryonic stem cells. Proc Natl Acad Sci USA.

[30] Fridrichova I, Kalinkova L, Karhanek M, Smolkova B, Machalekova K, Wachsmannova L (2021). miR-497-5p decreased expression associated with high-risk endometrial cancer. Int J Mol Sci.

[31] Liu J, Wang X, Song M, Du J, Yu J, Zheng W (2020). MiR-497-5p regulates osteo/odontogenic differentiation of stem cells from apical papilla via the Smad signaling pathway by targeting Smurf2. Front Genet.

[32] Xue WC, Chan KY, Feng HC, Chiu PM, Ngan HY, Tsao SW (2004). Promoter hypermethylation of multiple genes in hydatidiform mole and choriocarcinoma. J Mol Diagn.

[33] Lujambio A, Ropero S, Ballestar E, Fraga MF, Cerrato C, Setién F (2007). Genetic unmasking of an epigenetically silenced microRNA in human cancer cells. Cancer Res.

[34] Guil S, Esteller M (2009). DNA methylomes, histone codes and miRNAs: tying it all together. Int J Biochem Cell Biol.

[35] Zhang J, Yang C, Wu C, Cui W, Wang L (2020). DNA methyltransferases in cancer: biology, paradox, aberrations, and targeted therapy. Cancers(Basel).

[36] Shen S, Guo X, Yan H, Lu Y, Ji X, Li L (2015). A miR-130a-YAP positive feedback loop promotes organsize and tumorigenesis. Cell Res.

[37] Ngan HY, Bender H, Benedet JL, Jones H, Montruccoli GC, Pecorelli S, FIGO Committee on Gynecologic Oncology. (2003). Gestational trophoblastic neoplasia, FIGO 2000 staging and classification. Int J Gynaecol Obstet.

[38] Soslow RA, Dannenberg AJ, Rush D, Woerner BM, Khan KN, Masferrer J (2000). COX-2 is expressed in human pulmonary, colonic, and mammary tumors. Cancer.

[39] Zheng P, Chun Z, Wenjun Z, Chenchun W, Yi Z (2018). The STAT3/NFIL3 signaling axis-mediated chemotherapy resistance is reversed by Raddeanin A via inducing apoptosis in choriocarcinoma cells. J Cell Physiol.

