# Supplementary material for: miR-497-5p/SALL4 axis promotes stemness phenotype of choriocarcinoma and forms a feedback loop with DNMT-mediated epigenetic regulation
Source: Cell Death Dis. 2021 Nov 3;12(11):1046. doi: 10.1038/s41419-021-04315-1 (PMC8566582; doi:10.1038/s41419-021-04315-1)
Supplement: Supplementary file 1 — Supplementary Results. [file 41419_2021_4315_MOESM1_ESM.docx]

**Supplementary Results**

**Silencing miR-497-5p promotes stem-like characteristics in choriocarcinoma CSLCs.**

A continuous downregulation of miR-497-5p expression was observed in two generations of CSLCs (Supplementary Fig. 2A, *P*<0.05). Since miR-497-5p was inversely associated with SALL4 in choriocarcinoma tissues, we examined whether miR-497-5p downregulated CSLCs malignant phenotypes. Our results showed that silencing miR-497-5p significantly enhanced CSLCs self-renewal ability and stemness-marker expression (Supplementary Fig. 2B-D, *P*<0.05), together with colony formation enhancement, proliferation, drug-resistance, invasion and migration ability and VEGF and MMP-9 protein levels related to angiogenesis and metastasis, respectively (Supplementary Fig. 2E-J, *P*<0.01). In contrast, miR-497-5p overexpression reduced CSLCs stem-like malignant phenotypes (Supplementary Fig. 2E-J, *P*<0.05). Thus, these data indicated that miR-497-5p reduction played a crucial role in choriocarcinoma CSLCs development.
