# Supplementary figures and images for: miR-497-5p/SALL4 axis promotes stemness phenotype of choriocarcinoma and forms a feedback loop with DNMT-mediated epigenetic regulation
Source: Cell Death Dis. 2021 Nov 3;12(11):1046. doi: 10.1038/s41419-021-04315-1 (PMC8566582; doi:10.1038/s41419-021-04315-1)

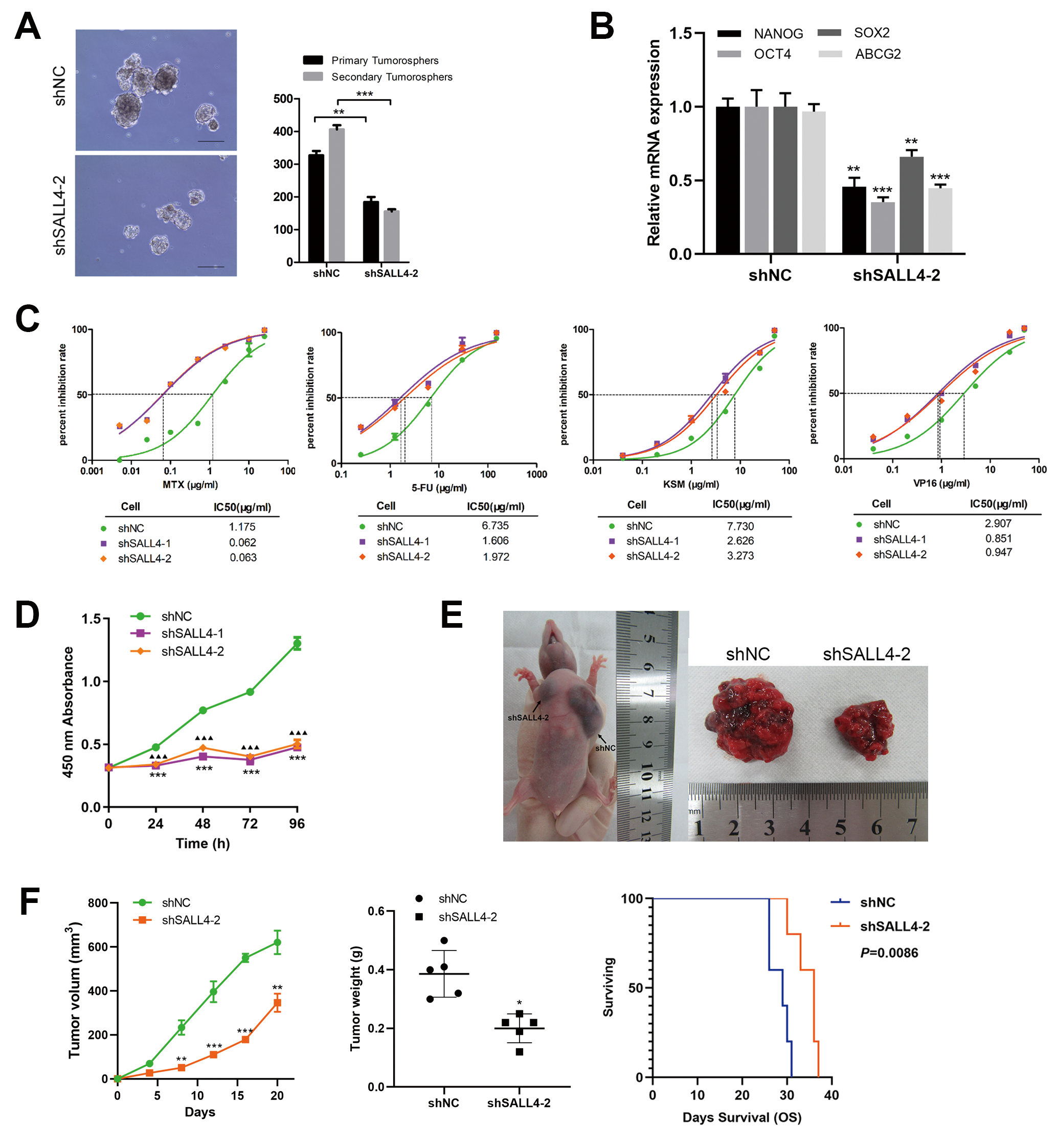

Supplement: Supplementary file 2 — Supplementary Figure 1. [file 41419_2021_4315_MOESM2_ESM.tif]

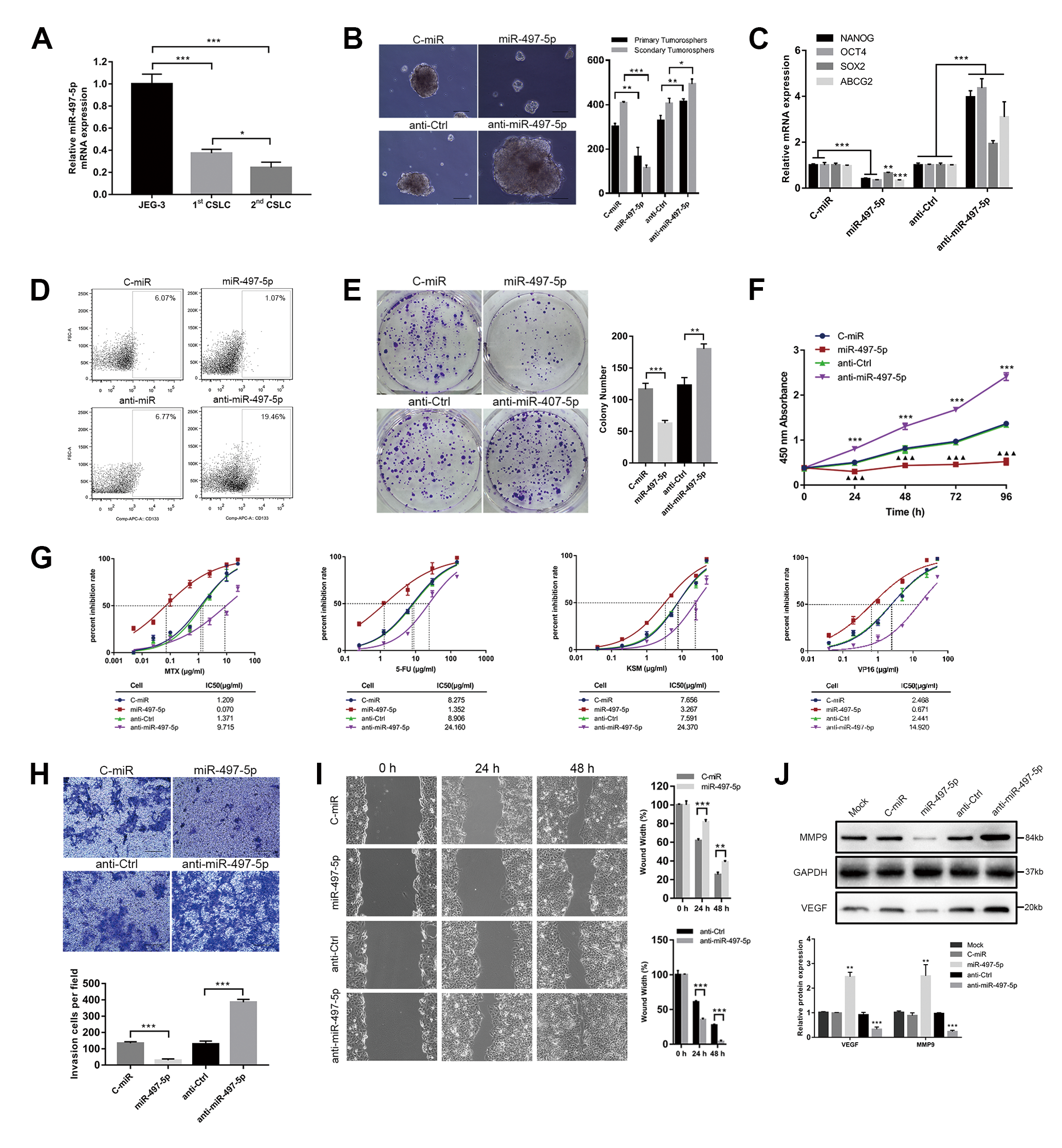

Supplement: Supplementary file 3 — Supplementary Figure 2. [file 41419_2021_4315_MOESM3_ESM.tif]

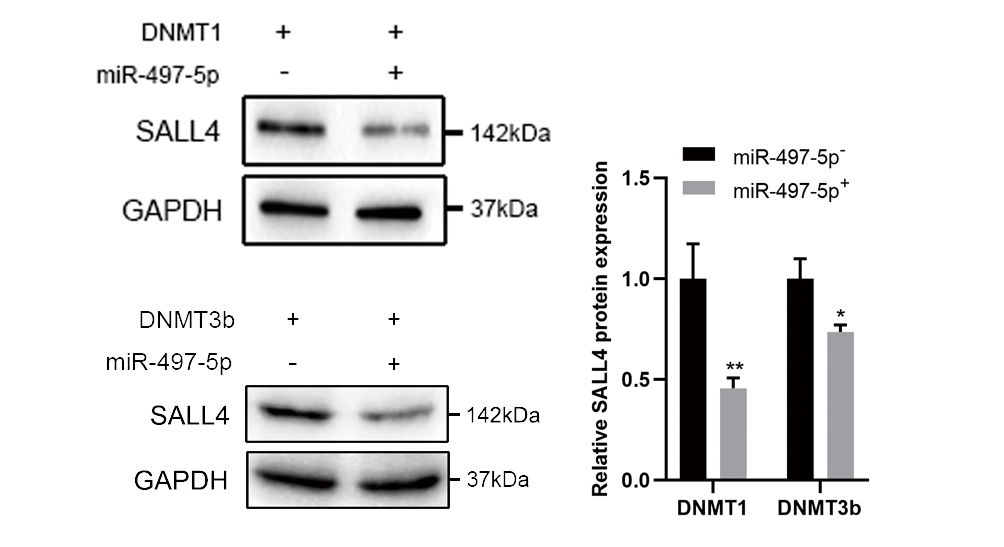

Supplement: Supplementary file 4 — Supplementary Figure 3. [file 41419_2021_4315_MOESM4_ESM.tif]
