# Supplementary material for: miR-497-5p/SALL4 axis promotes stemness phenotype of choriocarcinoma and forms a feedback loop with DNMT-mediated epigenetic regulation
Source: Cell Death Dis. 2021 Nov 3;12(11):1046. doi: 10.1038/s41419-021-04315-1 (PMC8566582; doi:10.1038/s41419-021-04315-1)
Supplement: Supplementary file 5 — Supplementary Figure legend. [file 41419_2021_4315_MOESM5_ESM.docx]

**Supplementary Figure 1** **shSALL4-2 reduces choriocarcinoma CSLCs malignant phenotype.** **A** Representative pictures (left, magnifications: ×100) of spheres and counts (right) of two generations. **B** Stemness genes expression by qRT-PCR analysis. **C** IC50 dose response curves of methotrexate, fluorouracil, dactinomycin and etoposide. **D** OD values of shSALL4 cells compared with control cells on proliferation ability for 96 h by CCK-8 assay (*shSALL4-1 vs. shNC; ▲shSALL4-2 vs. shNC). **E** Growth of subcutaneous xenograft tumors for following groups of shNC and shSALL4-2. **F** Tumor volumes were measured every four days for three weeks (the left). Tumor weight in the indicated mice (the middle). Kaplan-Meier curves of shSALL4-2 mice, compared with the control (the right). Data represent mean±SD from at least three independent experiments. *P<0.05, **P<0.01, ***P<0.001.

**Supplementary Figure 2 Silencing miR-497-5p promotes stem-like characteristics in choriocarcinoma CSLCs. A** qRT-PCR analysis of miR-497-5p levels between JEG-3 and CSLCs generations. **B** Representative pictures (left, magnifications: 🞨100) of spheres and counts (right) of two generations. **C** Stemness genes expression by qRT-PCR analysis. **D** Flow cytometry plots of CD133 cell surface expression. **E** Colony formation assays using single CSLCs (left) and colony number counts (right). **F** OD values of miR-497-5p or anti-miR-497-5p cells compared with control cells on proliferation ability for 96 h (*anti-miR vs. anti-Ctrl; ^▲^miR vs. C-miR). **G** IC_50_ dose response curves of methotrexate, fluorouracil, dactinomycin and etoposide. **H** Representative images (top, magnifications: 🞨100) and quantification (bottom) of invaded cells using transwell assay. **I** Migration images (left, magnifications: 🞨100) and quantification (right) of width area by wound healing assay. **J** Representative images (top) and quantification (bottom) of the effects of miR-497-5p over-expression or silencing on VEGF and MMP-9 levels by western blotting. Data represent mean±SD from at least three independent experiments. **P*<0.05, ***P*<0.01, ****P*<0.001.

**Supplementary Figure 3** **Overexpressed miR-497-5p can rescue the effect of DNMTs on SALL4.** Western blot analysis of SALL4 protein levels (left) and quantification (right) with overexpressed DNMT1/3b with or without overexpressed miR-497-5p transfection treatment. Data represent mean±SD from at least three independent experiments. **P*<0.05, ***P*<0.01.
