# Supplementary material for: miR-497-5p/SALL4 axis promotes stemness phenotype of choriocarcinoma and forms a feedback loop with DNMT-mediated epigenetic regulation
Source: Cell Death Dis. 2021 Nov 3;12(11):1046. doi: 10.1038/s41419-021-04315-1 (PMC8566582; doi:10.1038/s41419-021-04315-1)
Supplement: Supplementary file 6 — Supplementary Tables. [file 41419_2021_4315_MOESM6_ESM.doc]

**Supplementary Table 1. SALL4 expression and clinicopathological features in 36 patients**

| **Clinical**  **characteristic** | **Number of patients** | **Number of patients** | | ***P* Value** |
| --- | --- | --- | --- | --- |
|  |  | Low expression | High expression |  |
| Age (year) |  |  |  |  |
| <35 | 17 | 10 | 7 | 0.709 |
| ≥35 | 19 | 10 | 9 |  |
| Patient types  *(specimen location)* |  |  |  |  |
| Artificial abortion  *(normal villus)* | 10 | 9 | 1 | 0.017 |
| Choriocarcinoma | 26 | 12 | 14 |  |
| Untreated group  *(uterine cavity)* | 12 | 9 | 3 | 0.006 |
| **Refractory group**  ***(uterine cavity)*** | **14** | **3** | **11** |  |

**Supplementary Table 2. Oligonucleotides and primer sequences.**

| Gene | Primer Sequence |
| --- | --- |
| *SALL4* | Forward: 5’- TCGATGGCCAACTTCCTTC -3’  Reverse: 5’- GAGCGGACTCACACTGGAGA -3’ |
| *DNMT1* | Forward: 5’- CAGGAAGAACGGCCGCAGCA -3’  Reverse: 5’- AGGCTTTGCCGGCTTCCACG -3’ |
| *DNMT3a* | Forward: 5’- CAGTGCAGGTGACGAACATT -3’  Reverse: 5’- TGTTCCACCACACCTGTTTTGA -3’ |
| *DNMT3b* | Forward: 5’- GGCAAGTTCTCCGAGGTCTCTG -3’  Reverse: 5’- TGGTACATGGCTTTTCGATAGGA -3’ |
| *NANOG* | Forward: 5’- AGAACTCTCCAACATTCCTGAACCT -3’ |
|  | Forward: 5’- TGCCACCTCTTAGATTTCATTCTCT -3’ |
| *OCT4* | Forward: 5’- CTTGCTGCAGAAGTGGGTGGAGGAA -3’ |
|  | Forward: 5’- CTGCAGTGTGGGTTTCGGGCA -3’ |
| *SOX2* | Forward: 5’- GGGAAATGGGAGGGGTGCAAAAGA -3’ |
|  | Forward: 5’- TTGCGTGAGTGTGGATGGGATTGG -3’ |
| *ABCG2* | Forward: 5’- GCAAGATGTACTGGCGAAGA -3’ |
|  | Forward: 5’- CAGGTAGGCAATTGTGAGGAA -3’ |
| *GAPDH* | Forward: 5’- GCATGGCCTTCCGTGTTC -3’ |
|  | Forward: 5’- GATGTCATCATACTTGGCAGGTTT -3’ |
| *shSALL4-1* | TTCAACCTTGACATAGGTCGG |
| *shSALL4-2* | ATGTGCATCCGAATATGTTGC |
| *shDNMT1* | AACTCTCAAAGCCAGACTC |
| *shDNMT3b* | TTGCCATTCATGACAACAG |
