# Supplementary material for: miR-497-5p/SALL4 axis promotes stemness phenotype of choriocarcinoma and forms a feedback loop with DNMT-mediated epigenetic regulation
Source: Cell Death Dis. 2021 Nov 3;12(11):1046. doi: 10.1038/s41419-021-04315-1 (PMC8566582; doi:10.1038/s41419-021-04315-1)
Supplement: Supplementary file 7 — Supplementary Materials and methods. [file 41419_2021_4315_MOESM7_ESM.docx]

**Supplementary Materials and methods**

**Cell Transfection**

The overexpressed Letivector of miR-497-5p (miR-497-5p) and the inhibitor miR-497-5p Letivector (anti-miR-497-5p), including the two control vectors (C-miR and anti-Ctrl), were provided by Genechem (Shanghai, China). Cells were seeded in 6-well plates with a density of 2×10^5^ cells/well and transfected with lentivirus for 48 h and cultured with selection medium containing 8 μg/ml puromycin for 8 weeks to select the stable transfected cells. The LV-shSALL4, LV-DNMT1 and LV-DNMT3b, including the over-expressing lentiviral, were provided by Vigene Biosciences (Shandong, China).

**Cell proliferation assay**

Methotrexate (MTX), fluorouracil (5-FU), dactinomycin (KSM) and etoposide (VP16) were purchased from Sigma (Santa Clara, USA). Cell proliferation and the inhibitory concentration 50% (IC_50_) was explored with Cell Counting Kit-8 (Dojindo, Kumamoto, JPN) assay as per the protocols. Each well was added 10 µl CCK-8 for 2 h at 37℃. The absorbance was measured at 450 nm with a microplate reader (BioTek, Winooski, USA). The value of IC_50_ was calculated by Graphpad Prism 7 software.

**Western Blot Analysis**

Total proteins were extracted by RIPA lysis buffer (Beyotime biotechnology, Shanghai, China). Each sample (30-50 μg) were loaded onto an SDS-PAGE gel and transferred onto PVDF membranes (Merck, Darmstadt, DE). 5% milk was used for blocking the membrane for 1 h, and then incubated with various primary antibodies at 4℃ overnight with gently shaking. Secondary antibodies (CST, Darmstadt, DE) were added for 1h and detected with ECL reagent (Millipore, Billerica, USA), and exposed by chemiluminescence system (Syngene, Cambridge, UK). SALL4 was purchased from Abcam (Cambridge, UK). DNMT1, DNMT3b, VEGF and MMP-9 were purchased from CST (Darmstadt, DE). The gay value of each band was measured by Image J software. Data were normalized to GAPDH levels and were represented as relative expression levels compared with control group.

**Flow cytometry analysis**

Single cells were suspended in PBS and stained with anti-CD133/APC (BD Biosciences, USA) according to the manufacturer’s instructions. Samples were performed on the BD FACSCanto II (BD Biosciences, USA). IgG isotype was utilized to be controls. The data was analyzed by FlowJo software.

**Colony formation**

2000 cells with different treatments were seeded in 6-well plates. Changed the complete medium every other day for incubation. After 10-15 days, plates were stained with Crystal violet and the visible cell colonies were counted.

**Cell invasion assay**

Cell invasion ability was observed by a transwell assay. Matrigel (BD Biosciences, USA), diluted 1:2 in serum-free DMEM culture medium, was added to the upper chamber of a 24-well transwell plate. The transwell plates were incubated for 4-6 hours at 37℃. 5000 single cells were washed by PBS twice, suspended with 200 μL serum-free medium, and seeded in the upper chamber. 600 μL complete medium with 15% FBS was added to the lower chamber and incubated for 24 h at 37℃. Then the bottom of chamber was fixed with 4% paraformaldehyde for 20 min and stained with crystal violet. Five low-magnification (10x) fields were randomly selected and counted for the cell numbers. All experiments were performed in triplicate.

**Migration assay**

Cell migration ability was assessed by wound-healing assay. Cells were grown in 6-well plate and wounded with a sterile 10 μL pipette tip straightly. After cultured 0, 24 and 48 h, three randomly selected fields were recorded by microscope respectively. The wound area was examined by Image J software. The remaining extent of healing expressed as percent of the area between the original wound. The data on average of three measurements was available.

**Cell immunofluorescence**

JEG-3 cells were transfected with miR-497-5p or anti-miR-497-5p for 48 h. After transfection, cells were fixed with 4% paraformaldehyde and permeabilized with 0.1% Triton X-100. The nuclei of cells were stained with DAPI (blue). Cells were incubated with the SALL4 antibody overnight and then incubated with secondary antibody (red, Invitrogen, Carlsbad, CA) for 1 h. The cells were viewed under fluorescent microscope (Leica, Wetzlar, DE) within 2 hours. Five fields randomly selected and the Image J software was used to analyze the fluorescence intensity. The relative SALL4 level was represented by the relative average fluorescence intensity compared with the control group.

**Luciferase reporter assay**

The SALL4 promoter including two wild miR-497-5p binding sites or mutant type were cloned into the pGL3-Basic vector (Ribobio, Guangzhou, China). The luciferase reporter assay (Promega, Madison, USA) executed following the luciferase reporter assay protocol. 293T cells were co-transfected with reporter plasmid (pGL3-SALL4wt/mt), Vector or miR-497-5p as indicated. The fluorescence value was detected by GloMax-multimode reader (Promega, Madison, USA).

**Co-immunoprecipitation**

The total protein was lysed in RIPA lysis buffer, and then was incubated with anti-SALL4, DNMT1, DNMT3b. IgG as a negative control. Added to protein A/G agarose shaken for 4 h at 4℃ and centrifuged for 5 min at 2500 g following. The washed precipitation was suspended in SDS buffer with primary antibodies against SALL4, DNMT1 and DNMT3b, used for western blot analyses.

**Statistical analysis**

All data are expressed as means ± SD. Differences were determined by one-way ANOVA test and Chi-Square test. The data which not conform to the homogeneity of variance was calculated using Kruskal-Wallis test. Survival curves were plotted by the Kaplan-Meier method and compared by log-rank test. A probability of *P*<0.05 was considered statistically significant. All statistical analyses were performed using SPSS 22.0 software.
