# Supplementary material for: miR-497-5p/SALL4 axis promotes stemness phenotype of choriocarcinoma and forms a feedback loop with DNMT-mediated epigenetic regulation
Source: Cell Death Dis. 2021 Nov 3;12(11):1046. doi: 10.1038/s41419-021-04315-1 (PMC8566582; doi:10.1038/s41419-021-04315-1)
Supplement: Supplementary file 8 — Supplementary material 1. [file 41419_2021_4315_MOESM8_ESM.docx]

**Supplementary material 1**

The predicted target sites of seven miRNAs were considered to be best-supported based on the following criteria: (i) Due to the solid statistical approach, miRNAs with P_CT_ score > 0.7 for a single site in the Targetscan dataset were chosen. (ii) The top 20 miRNAs according to the target rank in miRDB dataset and the top 20 miRNAs according to the sum of mirSVR scores rank in miRanda dataset were chosen. (iii) miRNAs were predicted by the overlap of the three datasets results.
